# Necrotising Fasciitis of Head and Neck in Infants

**DOI:** 10.1007/s12070-020-01992-w

**Published:** 2020-07-28

**Authors:** Anjan kumar Sahoo, Ishwar Singh, Sunita Dhakal, Ashish Gopal

**Affiliations:** Dept. of ENT and Head & Neck Surgery, MAMC & LNJP Hospital, Delhi, India

**Keywords:** Necrotising, Fasciitis, Infants, Head and neck

## Abstract

Necrotising fasciitis (NF) is a rapidly progressing soft tissue infection having high risk of morbidity and mortality. Though it is a common condition encounter in surgical practice for adults, it’s incidence in children are very low. In children also abdomen is the most common site for NF. It’s involvement in head and neck is extremely rare. Here we discuss the clinical features and management of three cases of NFs in head and neck region of infants and also look for the etiology, clinical presentation and management for same.

## Introduction

Necrotising fasciitis (NF) is a rare, rapidly progressive, bacterial infection primarily involving the subcutaneous tissue and fascia with thrombosis of the subcutaneous blood vessels. The infection spreads rapidly through the fascial planes. Mostly caused by toxin producing virulent bacteria such as Group A streptococcus, enterobacteriaceae and non-group A streptococci. Vibrio and Aeromonas species and bacteroides are also responsible to some extent. Hippocrates was the first to recognise Necrotising fasciitis in 500 BC [1]. Wilson [2] proposed the term necrotising fasciitis in year 1950 from his observation that fascial necrosis is a constant feature of the syndrome not the cutaneous gangrene. The global prevalence of necrotising fasciitis is around 0.40 cases per 100,000 population [3]. It is very rare in children and particularly in infants. The incidence of NF in children is around 0.08 per 100,000 population. On the other hand in children, it has a fulminant course [4]. There was hardly any mention about the disease in pediatric textbooks before 1973 [5]. Although the disease affects all age groups but middle aged and elderly patients are very much prone for NF [6]. Here we describe three case of necrotising fasciitis of head and neck region in infants.

## Case 1

**Fig. 1:**
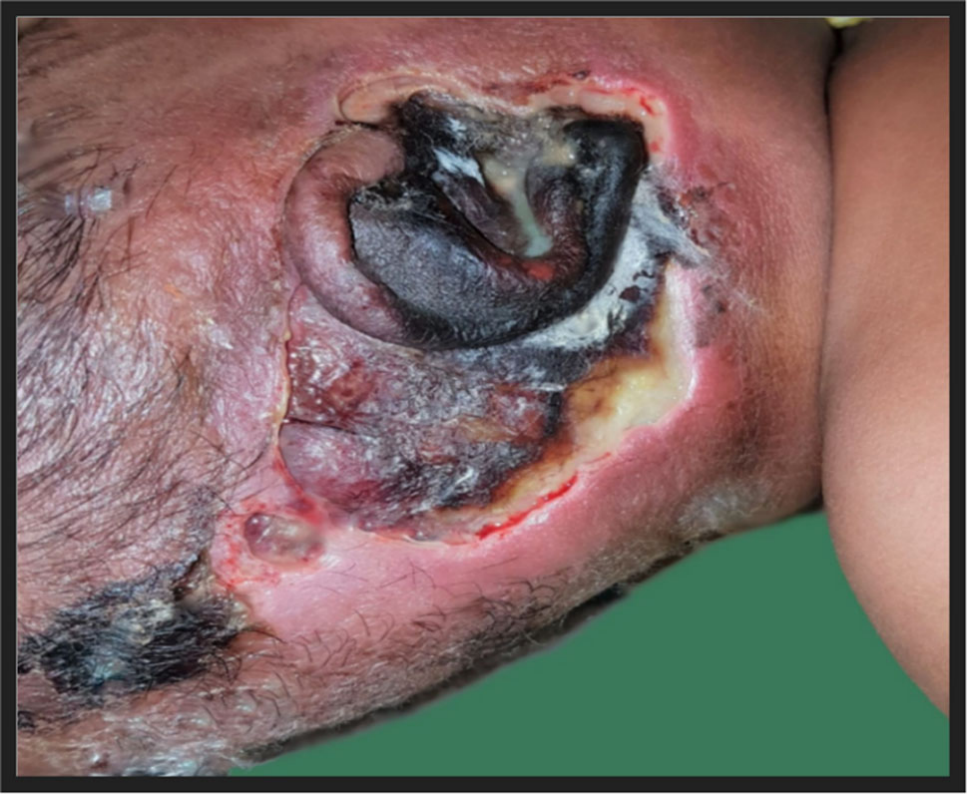
Blackish discolouration of right pinna surrounded by multiple erythematous pustules with fluctuant swelling over right temporo-parietal region

Four months female child presented with swelling over right pinna and fever for 10 days, history of pus discharge for 5 days. On examination there was blackish discolouration of the overlying skin of right pinna, postaural region and right temporal region with oozing of foul smelling discharge. The swelling was fluctuant with multiple erythematous pustules present around it (Fig. 1). He was having haemoglobin 11.4 gm/dl and pus culture suggestive of MRSA.

## Case 2

**Fig. 2:**
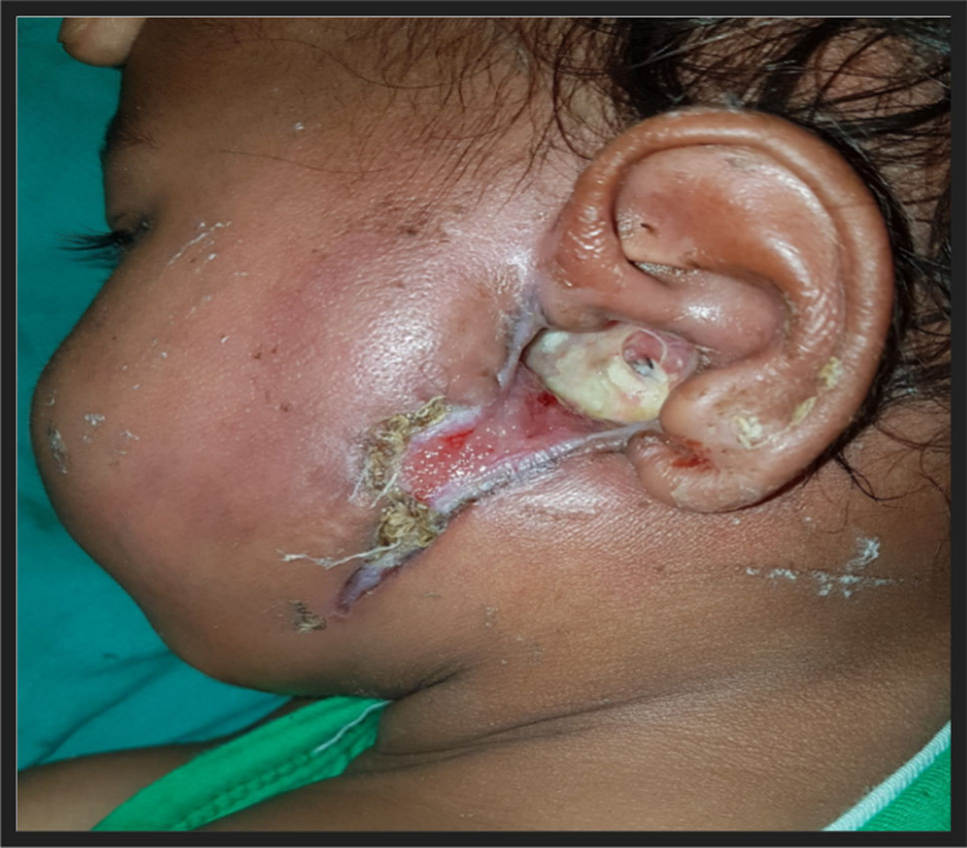
Erythematous swelling over left pre-auricular area surrounded by focal erosion of the cartilaginous EAC with the accumulation of purulent discharge and skin loss

Eight months male child presented with swelling over left pre auricular region for 8 days with history of left ear discharge. He was having high grade fever since last 5 days. On examination there was an erythematous swelling over left pre-auricular area surrounded by focal erosion of the cartilaginous external auditory canal with the accumulation of purulent discharge and skin loss (Fig. 2). He was having haemoglobin 9.9 gm/dl and pus culture suggestive of Pseudomonas aeruginosa.

## Case 3

**Fig. 3:**
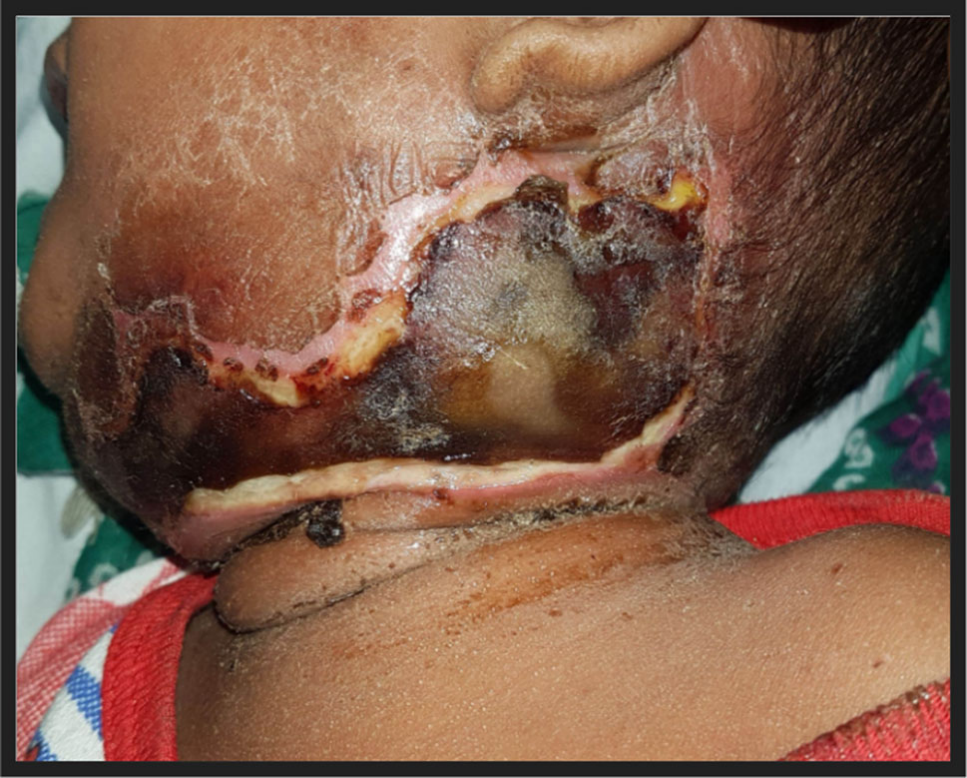
Erythematous swelling present over the anterior neck a/w blackish discolouration of the overlying skin and subcutaneous tissue

Nine months male child presented with swelling over neck since last 14 days. He was having fever since last seven days. On examination there was erythematous swelling present over the anterior neck with blackish discolouration of the overlying skin and subcutaneous tissue (Fig. 3). He was having haemoglobin 10.5 gm/dl and pus culture suggestive of *Pseudomonas aeruginosa*.

There is no history of any insect bite, trauma, drug allergy and all three were immunized as per the national programme schedule. All three had raised total leucocyte count and no growth was found in blood and urine culture.

## Management

**Fig. 4:**
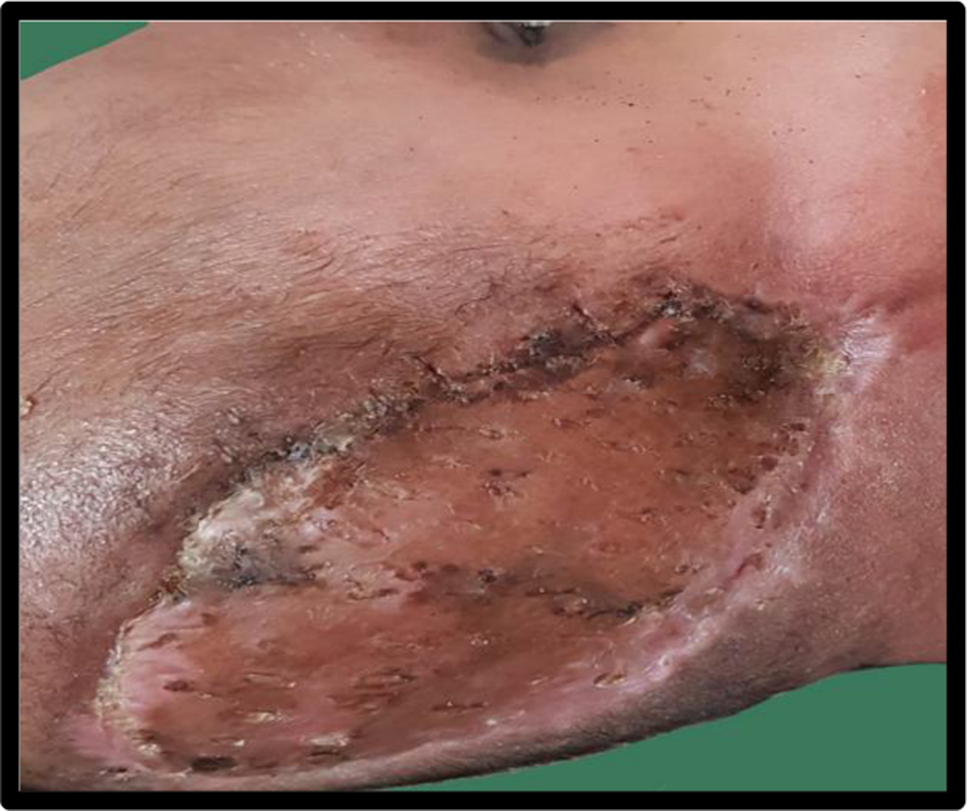
Case-1 post operative picture

**Fig. 5:**
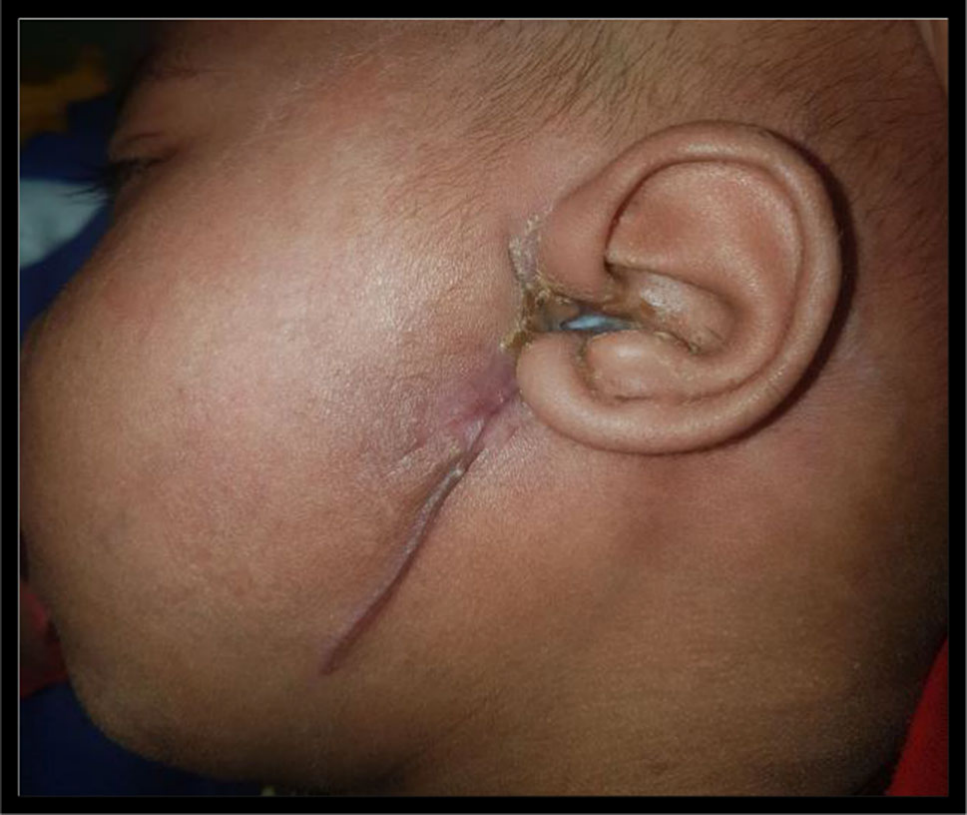
Case 2 wound healing done by secondary intention

**Fig. 6:**
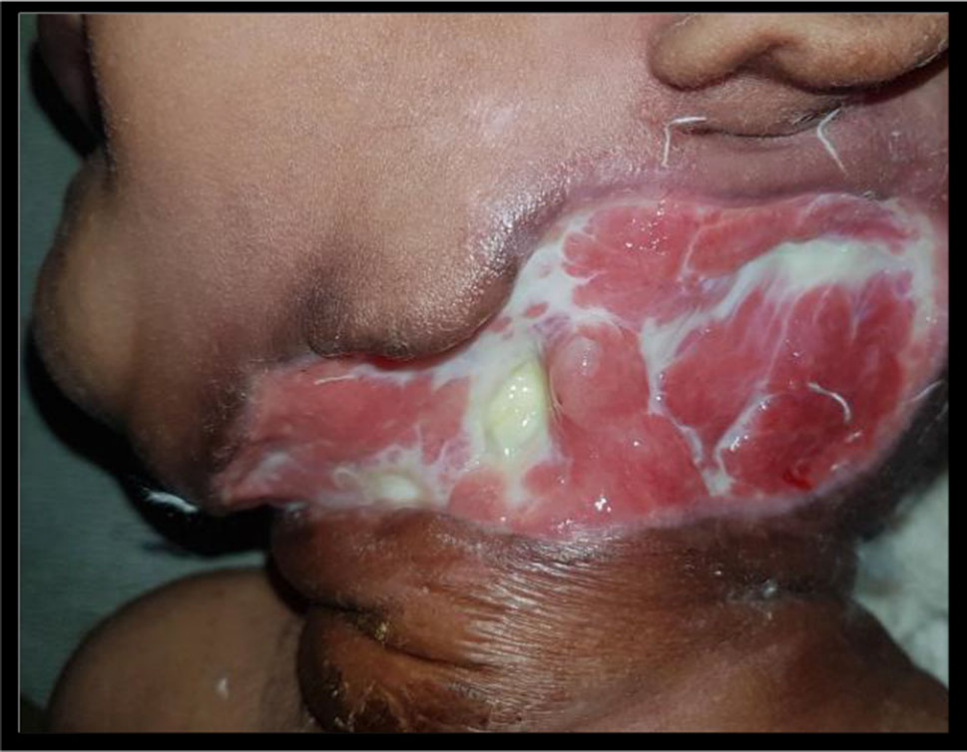
Case 3 postdebridement 20 days ready for grafting

Debridement of the necrosed skin and subcutaneous tissue of the involved area was done under general anaesthesia until healthy margins were achieved. All three were put on intravenous antibiotics according to pus culture and sensitivity. Daily dressings was done. After improvement of the general condition, spilt skin grafting of the defect was done except case-2 which was healed by secondary intention (Figs. 4, 5, 6).

## Discussion

We have focused our discussion of necrotizing fasciitis in infants only. Though lots of literature of necrotising fasciitis in adults are available, the knowledge is very scarce for children, particularly for infants. The most common site of involvement in paediatric patients is the abdominal wall followed by the thorax, back, scalp and extremities. There is no gender variation but occurs slightly more often in males [7]. It has a seasonal variation with higher incident in winter months [8]. Usually there is the presence of anaerobic bacteria that proliferate in a hypoxic environment and produce gas and accumulate in soft tissue spaces [9]. Tissue damage and systemic toxicity are believed to result from the release of endogenous cytokines and bacterial toxins. The common underlying predisposing factor for NF in children are omphalitis, mammilitis, balanitis, fetal scalp monitoring, post surgical complication, necrotising entero colitis, bullous impetigo, maternal mastitis, septicemia, immunodeficiency [10]. Fournier’s gangrene [11], Meleney’s gangrene [12], Phagadena gangrenosum, Hemolytic streptococcal gangrene, Flesh eating bacteria, Hospital gangrene are the synonyms of Necrotising fascitis described in accordance with it’s location in different region of body. Goldberg et al [13] found only 14 reported cases of NF in infants mostly resulting from circumcision or fetal scalp monitoring. Some reports also suggests the association of Haemophilus influenza type b and Chickenpox with NF [14, 15]. Fournier’s syndrome of six children aged between 3 and 12 weeks were reported by Adeyokunnu [16] where he suspected the portal of entry may be circumcision, diaper rash or may be perianal skin abscess. Necrotising enterocolitis and urachus anomalies [17] may also be responsible for NF. History of trauma which might be very minor, insect bite, unnoticed minor abrasions and lacerations may lead to development of NF [18]. Dental infections, tooth extractions, peritonsillar abscess, fracture of maxilla and mandible are some of the initiating factor for cervicofascial necrotizing fasciitis [19, 20].

Cellulitis, fever, tachycardia, swelling, skin discolouration, blistering, pain are the common clinical features of NF. Progression of the inflammation is terribly rapid. Severe sepsis, disseminated intravascular coagulation and multiple-organ failure are the late manifestation of NF.

Hundred years ago Meleny [21] recognized the importance of early surgical intervention, according to him surgery should not be delayed an hour after the diagnosis of NF has been made. At the same time one should not divert from the basic protocols like primary resuscitation and control of infection. All the discoloured, dead and necrotic tissue should be desloughed. Debridement of necrotic material must be continued until the skin and subcutaneous tissue can no longer be separated from the deep fascia [[2, 22]]. Every patients require thorough enteral or parenteral nutritional support, along with steroids and application of regional antibiotic.

## Conclusion

Neonatal NF is an uncommon but often deadly bacterial infection of the skin, subcutaneous tissue. It is distinguished by obvious tissue edema, necrosis of tissue and signs of systemic toxicity. High index of suspicion, adequate antibiotics, aggressive surgical therapy and supportive care are the mainstays of management of neonatal necrotizing fasciitis.
